# Homogeneous MMR Deficiency Throughout the Entire Tumor Mass Occurs in a Subset of Colorectal Neuroendocrine Carcinomas

**DOI:** 10.1007/s12022-020-09612-7

**Published:** 2020-03-06

**Authors:** Christoph Fraune, Ronald Simon, Claudia Hube-Magg, Georgia Makrypidi-Fraune, Martina Kluth, Franziska Büscheck, Tania Amin, Fabrice Viol, Wilfrid Fehrle, David Dum, Doris Höflmayer, Eike Burandt, Till Sebastian Clauditz, Daniel Perez, Jakob Izbicki, Waldemar Wilczak, Guido Sauter, Stefan Steurer, Jörg Schrader

**Affiliations:** 1 Institute of Pathology, University Medical Center Hamburg-Eppendorf, Martinistr. 52, 20246 Hamburg, Germany; 2 General, Visceral and Thoracic Surgery Department and Clinic, University Medical Center Hamburg-Eppendorf, Hamburg, Germany; 3 I. Medical Department – Gastroenterology and Hepatology, University Medical Center Hamburg-Eppendorf, Hamburg, Germany

**Keywords:** Microsatellite instability, Tissue microarray, Neuroendocrine tumor, Neuroendocrine carcinoma

## Abstract

Neuroendocrine neoplasms comprise a heterogeneous group of tumors, categorized into neuroendocrine tumors (NETs) and neuroendocrine carcinomas (NECs) depending on tumor differentiation. NECs and high-grade NETs (G3) confer a poor prognosis, demanding novel treatment strategies such as immune checkpoint inhibition in tumors with microsatellite instability (MSI). To study any possible intratumoral heterogeneity of MSI, a tissue microarray (TMA) containing 199 NETs and 40 NECs was constructed to screen for MSI using immunohistochemistry (IHC) for the mismatch repair (MMR) proteins MLH1, PMS2, MSH2, and MSH6. Four cases suspicious for MSI were identified. Validation of MSI by repeated IHC on large sections and polymerase chain reaction (PCR)–based analysis using the “Bethesda Panel” confirmed MSI in 3 cecal NECs. One pancreatic NET G3 with MSI-compatible TMA results was MMR intact on large section IHC and microsatellite stable (MSS). The remaining 235 tumors exhibited intact MMR. Protein loss of MLH1/PMS2 was found in two and MSH6 loss in one cancer with MSI. Large section IHC on all available tumor-containing tissue blocks in NECs with MSI did not identify aberrant tumor areas with intact MMR. Our data indicate that MSI is common in colorectal NECs (3 out of 10) but highly infrequent in neuroendocrine neoplasms from many other sites. The lack of intratumoral heterogeneity of MMR deficiency suggests early development of MSI during tumorigenesis in a subset of colorectal NECs and indicates that microsatellite status obtained from small biopsies may be representative for the entire cancer mass.

## Introduction

Neuroendocrine neoplasms occur at various organ sites and comprise a heterogeneous group of epithelial tumors ranging from well-differentiated neuroendocrine tumors (NETs) to poorly differentiated carcinomas, called neuroendocrine carcinomas (NECs). NETs clinically often present as low-grade malignancies, while NECs behave more aggressively with rapid disease progression and poor long-term survival, demanding for novel treatment options.

Microsatellite instability (MSI) refers to a distinctive pattern of increased mutational load in certain tumors, typically caused by a defective DNA mismatch repair (MMR) apparatus, and is characterized by accumulated length variations of repetitive DNA sequences (microsatellites) throughout the genome. Protein loss of at least one of the major components of the MMR system—MLH1, PMS2, MSH2, or MSH6, which can easily be analyzed by immunohistochemistry (IHC)—provides strong indirect evidence for MSI. MSI can also be detected via direct analysis of a predefined panel of DNA microsatellite loci with classic polymerase chain reaction (PCR)–based methods or novel high-throughput molecular techniques [1]. Favorable response rates for immune checkpoint inhibitors in cancers with MSI have dramatically increased the clinical request for MSI testing, even in tumor types with low expected rates of MSI. This was paralleled by the 2017 site-agnostic FDA approval of the PD-1 antibody pembrolizumab for advanced cancers with MMR deficiency/MSI-high.

Data on MSI in neuroendocrine neoplasms are limited. Recent studies have found tumors with MSI among neuroendocrine carcinomas (some combined with a conventional adenocarcinoma component, MINEN) of the stomach, small intestine, and colorectum [2–6]. The few available studies describing MSI in cohorts of NETs are basically limited to pancreatic primaries with reported frequencies between 10% (5/48) and 33% (18/55) by PCR-based methods [7, 8]. Intratumoral heterogeneity of MSI—a potential strong confounder for molecular based treatments—has been observed in several cancer types, including colorectal cancer [9–13]. However, intratumoral heterogeneity of MSI in neuroendocrine neoplasms has not been evaluated so far.

To systematically assess potential intratumoral heterogeneity of MMR protein expression in neuroendocrine neoplasms with MMR deficiency, a cohort of 239 NETs and NECs was screened on a tissue microarray (TMA) format by IHC, followed by a large section evaluation of cancers suspected for MSI by repeated IHC and PCR analysis.

## Material and Methods

### Tissue Microarray

A TMA comprising 239 neuroendocrine neoplasms and various control tissues was constructed using one tissue core per tumor from archived material from patients diagnosed with a NET or NEC at the University Medical Center Hamburg-Eppendorf between 2009 and 2018. TMA construction was described earlier [14]. Clinical and pathological information were obtained from the patient’s medical records and reports. NET- and NEC-categorization was based on the criteria described in the recent 5th edition of the WHO classification of tumors of the digestive system, and differentiation between NECs and high-grade (G3) NETs was adopted from Tang et al. [15]. No mixed tumors with an additional non-neuroendocrine tumor component (MINEN) were identified. Utilization of archived diagnostic leftover tissues for manufacturing of TMAs and their analysis for research purposes as well as patient data analysis has been approved by local laws (HmbKHG, §12,1) and by the local ethics committee (Ethics commission Hamburg, WF-049/09). All work has been carried out in compliance with the Helsinki Declaration.

### Immunohistochemical Analyses

To screen for MSI cancers, freshly taken TMA sections were immunostained on 1 day and in one experiment using an automated immunostainer (Dako/Agilent Autostainer Link 48). Primary antibody specific for MLH1 (clone ES05, mouse), PMS2 (clone EP51, rabbit), MSH2 (clone FE11, mouse), and MSH6 (clone EP49, rabbit) (all Ready-to-Use, all from DAKO, Glostrup, Denmark) was applied for 20 min (MLH1, MSH2, MSH6) or 30 min (PMS2). Bound antibody was visualized using the EnVision Kit (Dako, Glostrup, Denmark) according to the manufacturer’s directions. Tumors were scored as negative (0) when nuclear staining was absent in all captured tumor cells and positive (+) when unequivocal nuclear staining in tumor cells (irrespective of staining intensity or percentage of stained cells) was observed. In spots showing a negative (0) result for the tumor cells, presence (+) or absence (−) of nuclear staining in peritumoral stromal or inflammatory cells was also recorded (internal control). For TMA spots with a staining loss of any MMR protein in tumor cells but not in stromal/inflammatory cells (suspected MSI), IHC was repeated on a corresponding large section of the routinely archived tumor material and PCR analysis was performed. In case of non-interpretable TMA spots (lack of unequivocal tumor tissue or negative (0) tumor cells accompanied by absence of an internal control (−)), staining was repeated on a representative large section.

### PCR Analysis

For all cases with suspected MSI based on TMA screening, a fluorescent PCR-based assay was performed (MSI Analysis System; Promega, Madison, WI), incorporating the five microsatellite loci of the “Bethesda Panel” which includes two mononucleotide repeats (BAT25, BAT26) and three dinucleotides repeats (D2S123, D5S346, and D17S250) [16]. Analysis was based on DNA extracted from tumor tissue that was dissected from a large section of the respective tumor block corresponding to the TMA case and from non-neoplastic control tissue of the patient. Percentage of tumor cells was at least 50% within the analyzed tissue area. Status of MSI-high was given when at least 2 (≥ 40%) of the markers showed instability (i.e., length variation compared with control tissue) and status of MSI-low if one of the analyzed loci showed instability; otherwise, microsatellite stability (MSS) was assumed.

### Evaluation of Cancer Heterogeneity

If MSI was confirmed, all available archived tumor-containing tissue blocks were analyzed to search for intratumoral heterogeneity. All together 20 large sections were analyzed for 3 patients with confirmed MMR deficiency.

## Results

### TMA Screening and Large Section Validation

**Fig. 1:**
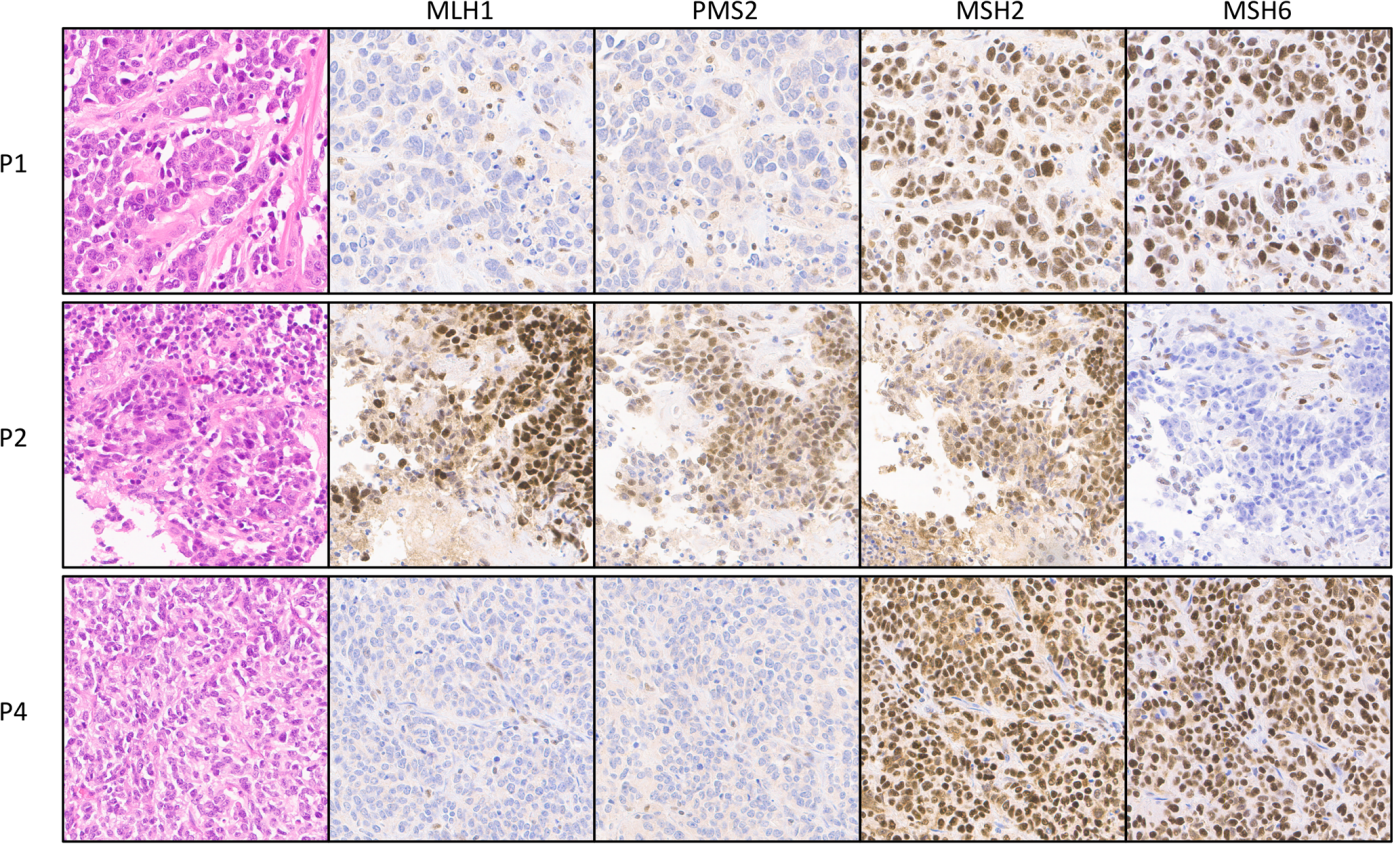
TMA spots of the three colorectal NEC with MSI. Two cancers showed loss of MLH1/PMS2 and one cancer isolated loss of MSH6

From 239 neuroendocrine neoplasms, including 199 NETs and 40 NECs of gastroenteropancreatic and pulmonary sites (Table 1), TMA screening obtained interpretable results in 224 (94%) cases. Tumors were considered interpretable if either unequivocal loss of staining for at least one of the four MMR proteins MLH1, PMS2, MSH2, or MSH6 with an internal positive control (MMR deficient) or unequivocally retained expression for all 4 MMR proteins (MMR intact) was observed. For non-interpretable TMA cases—due to lack of unequivocal tumor tissue on the TMA spot (*n* = 15)—large section IHC was performed and revealed intact MMR in each cases. Two hundred twenty from the 224 tumors with interpretable results from TMA screening were also MMR intact, resulting in 235 of 239 neuroendocrine neoplasms demonstrating retained expression of all four MMR proteins. The remaining 4 tumors were all high-grade neuroendocrine neoplasms, including one pancreatic NET G3 and 3 colorectal NECs, demonstrating loss of one or two MMR proteins on the TMA accompanied by an adequate internal positive control, and were thus considered suspicious for MSI (Table 2). Representative micrographs from the three cecal NECs with MMR deficiency are shown in Fig. 1.

**Table 1:** Study cohort of neuroendocrine neoplasms separated by anatomic site, tumor type and tumor grade

Site	NET			NEC		Sum
	G1	G2	G3	SC	LC	
Esophagus				1	4	5
Stomach	5	2			1	8
Duodenum	2	2				4
Ampulla Vateri	1					1
Jejunum	2	1				3
Meckel’s diverticulum	1	1				2
Ileum	36	17	1		1	55
Appendix	20	2				22
Colorectum	8	1		5	5	19
Pancreas	28	26	5	1	4	64
Gall bladder		1		3	1	5
Lung	12	7		2	5	26
Metastatic*	6	10	2	5	2	25
Sum	121	70	8	17	23	239
	199			40		

*Primary tumor sites include ileum (7), pancreas (2), lung (2), rectum (1), and unknown (CUP; 6) for metastatic NETs and lung (2), appendix (1), and unknown (CUP; 4) for metastatic NECs

**Table 2:** Summary of IHC and PCR data on neuroendocrine neoplasms with suspected MSI based on TMA screening. Not all of the 5 microsatellite loci of the “Bethesda Panel” were evaluable by PCR for each tumor with MSI; however, status of MSI was still unequivocal in each case

Tumor type	TMA screening				Large section validation						Entire tumor	
	MLH1	PMS2	MSH2	MSH6	MLH1	PMS2	MSH2	MSH6	Status IHC	PCR	Tumor blocks (*n* =)	MMR pattern
Pancreatic NET G3	+	−	+	+	+	+ (weak)	+	+	MMR intact	MSS (0/5)	−	−
Colorectal NEC	−	−	+	+	−	−	+	+	MMR deficient	MSI-high (4/4)	5	Homogeneous MMR deficiency
Colorectal NEC	+	+	+	−	+	+	+	−	MMR deficient	MSI-high (3/5)	7	Homogeneous MMR deficiency
Colorectal NEC	−	−	+	+	−	−	+	+	MMR deficient	MSI-high (3/3)	8	Homogeneous MMR deficiency

### MSI Validation

**Fig. 2:**
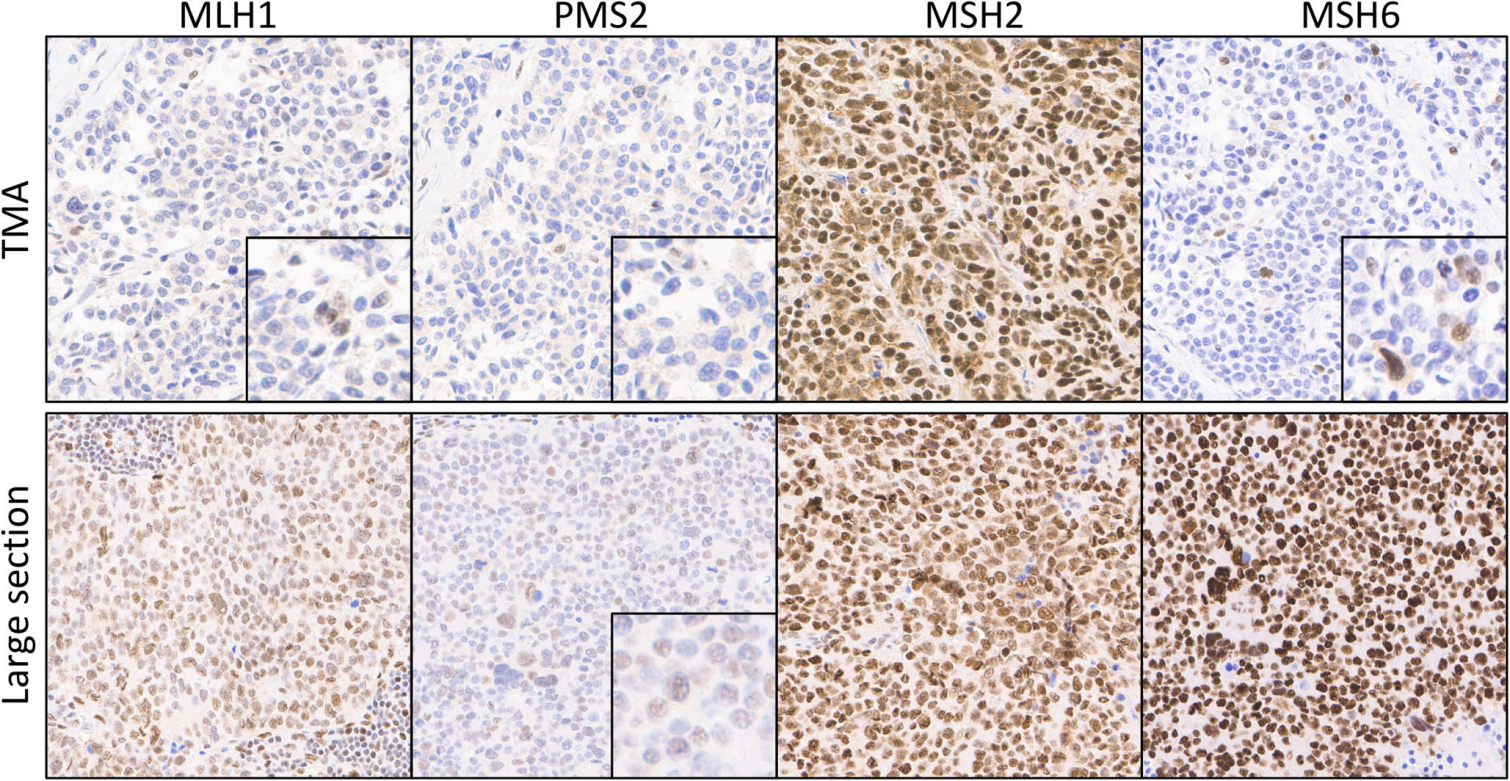
Suspected MMR deficiency by TMA screening in a NET with intact MMR. For one pancreatic NET G3, TMA immunohistochemistry was interpreted as isolated PMS2 loss but repeated large section IHC showed intact MMR and PCR revealed microsatellite stability (MSS)

Large section validation confirmed MMR deficiency in 3 of 4 suspected cases, all representing NECs of the colorectum (Table 2). Two of these cancers were negative for MLH1/PMS2 and one showed isolated protein loss of MSH6. All three were MSI-high by PCR-analysis, were located in the cecum, and demonstrated large cell phenotype. No non-neuroendocrine tumor component was found. Clinicopathological data did not reveal any further clear-cut associations as compared with colorectal NECs with intact MMR (Table 3). The one tumor with suspected MSI based on TMA screening that turned out MMR intact on large section analysis was a pancreatic NET G3 with strongly attenuated PMS2 immunoreactivity throughout the majority of the tumor, but PMS2 expression was still retained and the other analyzed MMR proteins were inconspicuous. A comparison with the PMS2 staining on a corresponding large section revealed that the TMA spot had unluckily been taken from an area with markedly diminished immunoreactivity (Fig. 2). PCR analysis did not show any instability among the analyzed microsatellite loci (MSS) in this tumor.

**Table 3:** MSI status and clinicopathological parameters in colorectal NECs

No.	MMR deficiency	Location	Age	Gender	Small cell/large cell	Mitoses (10 HPF)	Ki-67 (%)	CD56	Syn	Chromo	MINEN	TNM
1	Yes	Cecum	95	f	LC	57	50	+++, 100%	++, 100%	−	−	pT4, pN0
2	Yes	Cecum	59	m	LC	39	40	++, 100%	+++, 100%	+++, 100%	−	pT2, pN0, pM1 (HEP)
3	No	Right flexure	64	f	SC	85	90	++, 80%	++, 20%	−	−	pT4, pN1
4	Yes	Cecum	81	m	LC	52	90	+++, 100%	+, 40%	++, 60%	−	pT4a, pN0
5	No	Sigma	70	f	SC	120	80	+++, 100%	+++, 80%	+++, 5%	−	pT4a, pN2b
6	No	Transversum	54	m	LC	31	70	n.a.	+++, 100%	++, 60%	−	pT3, pN1, pM1 (PER)
7	No	Cecum	87	f	LC	105	80	+++, 20%	+++, 90%	−	−	pT3b, pN2b, pM1a (HEP)
8	No	Cecum	66	f	SC	145	70	n.a.	+++, 90%	+++, 90%	−	pT4, pN1
9	No	Ascendens	82	f	SC	104	90	+++, 90%	++, 70%	−	−	pT4b, pN2b, pM1 (HEP, PER)
10	No	Rectum	77	m	SC	87	80	n.a.	+++, 90%	−	−	pT4b, pN1b

### MSI Heterogeneity Analysis

In the 3 NECs with confirmed MSI, IHC for MLH1, PMS2, MSH2, and MSH6 from all available archived cancer-containing tumor blocks (*n*=20) revealed consistent MMR protein loss throughout the entire tumor mass. No concurrent tumor areas with intact MMR were found.

## Discussion

A TMA containing 199 NETs and 40 NECs of various primary sites was manufactured for the purpose of this study as a screening tool to identify tumors with MSI among the heterogeneous group of neuroendocrine neoplasms. Detection of rare events is an ideal application for TMAs. For example, earlier studies identified 42 tumors harboring IDH1 mutations by screening 15,531 prostate cancers (0.3%) or 43 tumors exhibiting CD117 overexpression in a cohort of 1654 breast carcinomas (2.6%) [17, 18]. We recently also identified MMR deficiency/MSI in 5 tumors in a series of 448 bladder cancers (1.1%), in 7 tumors in a series of 200 advanced and/or hormone refractory prostate cancers (3.5%), and in 9 tumors in a series of 479 ovarian cancers (1.8%) (unpublished data). In the present study, only one of the 4 tumors with suspected MMR deficiency/MSI based on TMA screening turned out to be MMR intact on subsequent large section analysis. The discrepant results between TMA and large section IHC were observed in a pancreatic NET G3 and attributable to suboptimal immunoreactivity of major tumor areas, which—for PMS2—resulted in still faintly detectable staining of stromal cells but negativity of tumor cells on the TMA spot in contrast to unequivocally retained MMR protein expression in cancer cells on large section. This observation may reflect somewhat higher PMS2 expression levels in a subset of stromal cells as compared with cancer cells.

Among the 239 analyzed neuroendocrine neoplasms, MSI was confirmed in 3 cases (1.3%), all representing NECs of the cecum, suggesting a considerable prevalence of MSI (30%; 3/10) in colorectal NECs, although this may not reflect the true MSI rate in colorectal NECs due to the small tumor cohort. None of the other primary NECs from different sites of the gastrointestinal tract (*n* = 15) and the lung (*n* = 7) exhibited MSI. These data fit with previous studies reporting particularly high rates of MSI in NECs of the colorectum. Sahnane et al. found MSI in 6 of 37 (16%), Olevian et al. in 2 of 29 (7%), Furlan et al. in 2 of 21 (10%), and La Rosa et al. in 3 of 35 (14%) colorectal NECs [2, 4–6]. In the study by Sahnane et al. also 4 tumors with MSI were identified among 36 gastric NECs (11%) [2]. A high frequency of MSI (44%) based on IHC detection of MMR protein loss was further observed by Pocrnich et al. in a cohort of 18 large cell NECs of the endometrium [19]. In contrast, NECs from other organs have rarely been found to exhibit MSI. Sahnane et al. found only one other tumor with MSI among 16 NECs of the duodenum, esophagus, pancreas, and gall bladder [2].

Taken together, the available data suggest that the prevalence of MSI in NECs is site-dependent and closely related to those organ sites where the exocrine neoplastic counterparts of NECs—adenocarcinomas—are also frequently affected by MSI, such as endometrial, colorectal, and gastric adenocarcinomas. All 3 colorectal NECs with MSI in the present study were located in the cecum, corresponding well to the strong association of MSI in sporadic colorectal adenocarcinomas with tumor location in the right colon [20]. A link between MSI in conventional and neuroendocrine carcinomas of distinct organ sites is further supported by several reports of MSI in NECs with concomitant adenocarcinoma components in the affected tumors (MINENs) [2, 4, 6]. The concept of site dependency of MSI in NECs is also supported by the reported absence of MSI-high tumors among 107 small cell lung cancers by Chung et al. [21], paralleling the low frequency of MMR deficiency in non-small cell lung cancer [22–26]. Based on the available data, MSI should be regularly tested in colorectal, gastric, and endometrial NECs. As NECs confer a very poor prognosis and no second line treatment after failure of Cis/Carboplatin and Etoposide has been established [27], MSI testing should be performed during initial diagnosis to avoid unnecessary treatment delay upon progression after first-line treatment. As colorectal NECs have less than 50% response rate upon Platin-based chemotherapy and an overall survival of less than 9 months, this patient group might in particular benefit from upfront MSI testing and initiation of immune checkpoint inhibitor therapy [27].

In contrast to NECs, none of the 199 NETs exhibited MSI in the present study. That MSI is highly infrequent in NETs fits well with data from earlier studies. MSI was not found in previous studies analyzing 56 rectal [28], 14 small bowel [29], 16 pancreatic [30], 29 gastroenteropancreatic [31], and 38 foregut/midgut [32] as well as 35 pancreatic and 34 small intestinal NETs [33] by PCR. In contrast, two earlier studies described relevant MSI rates in pancreatic NETs [7, 8]. However, as those studies were performed 10 and more years ago, the applied approach to identify or define MSI differs from current standards. House et al. reported MSI in 5 of 48 (10%) pancreatic NETs using the “Bethesda Panel,” but status of MSI was already given when merely the mononucleotide repeat locus BAT-25 was instable, contrasting the current cut-off requiring instability in 40% of loci to justify MSI [7]. Mei et al. reported MSI-high, defined as instability of at least 4 loci of an extended panel of 12 microsatellites, in 18 of 55 (33%) insulinomas [8]. In contrast, all 8 insulinomas among the cohort of 56 pancreatic NETs analyzed in the present study were MMR intact. There are, however, reports describing MMR deficiency in few individual cases of pancreatic NETs in patients with hereditary MMR deficiency (Lynch Syndrome) [34, 35].

Evaluation of the MMR status throughout all available cancer-containing tumor blocks revealed homogeneous MMR protein loss in all three NECs with confirmed MSI. This is in agreement with our previous observations of high homogeneity of MSI in prostate [36], ovarian [37], and bladder cancer (unpublished data). Overall, these data may suggest that MMR inactivation generally occurs early in tumorigenesis. Regarding treatment purposes, homogeneity of MSI reduces the risk that molecular parameters obtained from small biopsies may not be representative for the entire cancer mass, a potential strong confounder for individualized therapies. It is a limitation of our study that results for all tumors with intact MMR were derived from TMA cores and it cannot be completely ruled out that a tumor with heterogeneous MMR/MSI status has been missed among this group because a MMR-deficient tumor area has not been represented on the respective TMA spot.

PCR analysis revealed MSI-high in all 3 cases of MMR deficiency. Although the number of cases is too small to draw major conclusions, the high concordance between IHC and PCR results is not surprising. The “Bethesda Panel,” a selection of 5 specific mono- and dinucleotide repeats, has been developed based on data derived from colorectal cancers [16]. As different microsatellite loci are not equally affected by frameshifts in the state of MSI due to an association between transcriptional activity and the occurrence of instability in individual repeat loci [38, 39], a tissue-dependent pattern of MSI is conceivable. Loci typically demonstrating instability in colorectal adenocarcinomas with MSI, i.e., the loci covered by the “Bethesda Panel,” may be affected with comparable frequency in colorectal NECs exhibiting MSI, just as both tumor types certainly show genetic overlap due to their shared origin from large bowel mucosa.

In summary, the detection of MSI in 3 of 10 colorectal NECs but not in other neuroendocrine neoplasm suggests that MSI affects NECs of the colorectum in a relevant manner, similar to colorectal adenocarcinomas. In contrast, MSI is highly infrequent in NETs irrespective of tumor site. The observed complete homogeneity of MMR deficiency indicates that the use of minor biopsy samples should result in highly representative data and hints towards MSI as an early event in tumorigenesis. Testing for MSI early in the course of disease might open novel treatment options for colorectal NECs.
